# Molecular Foundations of Reproductive Lethality in *Arabidopsis thaliana*

**DOI:** 10.1371/journal.pone.0028398

**Published:** 2011-12-02

**Authors:** Rosanna Muralla, Johnny Lloyd, David Meinke

**Affiliations:** Department of Botany, Oklahoma State University, Stillwater, Oklahoma, United States of America; University of Calgary, Canada

## Abstract

The SeedGenes database (www.seedgenes.org) contains information on more than 400 genes required for embryo development in *Arabidopsis*. Many of these *EMBRYO-DEFECTIVE* (*EMB*) genes encode proteins with an essential function required throughout the life cycle. This raises a fundamental question. Why does elimination of an essential gene in *Arabidopsis* often result in embryo lethality rather than gametophyte lethality? In other words, how do mutant (*emb*) gametophytes survive and participate in fertilization when an essential cellular function is disrupted? Furthermore, why do some mutant embryos proceed further in development than others? To address these questions, we first established a curated dataset of genes required for gametophyte development in *Arabidopsis* based on information extracted from the literature. This provided a basis for comparison with *EMB* genes obtained from the SeedGenes dataset. We also identified genes that exhibited both embryo and gametophyte defects when disrupted by a loss-of-function mutation. We then evaluated the relationship between mutant phenotype, gene redundancy, mutant allele strength, gene expression pattern, protein function, and intracellular protein localization to determine what factors influence the phenotypes of lethal mutants in *Arabidopsis*. After removing cases where continued development potentially resulted from gene redundancy or residual function of a weak mutant allele, we identified numerous examples of viable mutant (*emb*) gametophytes that required further explanation. We propose that the presence of gene products derived from transcription in diploid (heterozygous) sporocytes often enables mutant gametophytes to survive the loss of an essential gene in *Arabidopsis*. Whether gene disruption results in embryo or gametophyte lethality therefore depends in part on the ability of residual, parental gene products to support gametophyte development. We also highlight here 70 preglobular embryo mutants with a zygotic pattern of inheritance, which provide valuable insights into the maternal-to-zygotic transition in *Arabidopsis* and the timing of paternal gene activation during embryo development.

## Introduction

Essential genes have long played an important role in microbial and medical genetics. In recent years, several factors have contributed to the establishment of large datasets of essential genes in microorganisms: the failure of mutant cells to proliferate is readily detected in high-throughput gene disruption experiments; the products of essential genes define promising targets for novel, antimicrobial compounds relevant to human health; and lethal mutations in essential genes help to define the minimal gene set required to sustain a living cell [1], [2]. Many laboratories have contributed to the large-scale identification of essential genes in bacteria [3]–[6] and yeast [7]–[10], and to the establishment of a database of essential genes for comparative studies [11]. Related efforts with *Drosophila* [12]–[14], *Caenorhabditis* [15], mouse [16], [17], and humans [18] have generated a wealth of information on the diversity of gene products required for viability in multicellular eukaryotes. Recent evolutionary studies have also explored the relationship between gene function, duplication, and essentiality in animal systems [19]–[23].

Our desire to establish a comparable dataset of essential genes in a model plant [24], [25] arose from a longstanding interest in the isolation and characterization of embryo-defective (*emb*) mutants of *Arabidopsis* [26]–[28]. Identifying the genes responsible for these mutant phenotypes was facilitated by advances in T-DNA insertional mutagenesis, which enabled large-scale, forward genetic screens for tagged, loss-of-function mutants defective in seed development [29], [30]. Eventually, this led to the establishment of the SeedGenes database (www.seedgenes.org), which presents detailed information on genes required for embryo development in *Arabidopsis* [24]. The December 2010 database release includes 402 *EMB* genes with an essential cellular function required to produce a normal, mature embryo. Many EMB proteins are likely to be required throughout the life cycle. Mutants exhibit defects in embryogenesis because that is when the absence of a functional gene product first becomes critical.

Other research groups have pursued a complementary approach to the analysis of essential genes in *Arabidopsis* through the isolation and characterization of mutants defective in gametophyte development [31]–[35]. Identification of these mutants has been facilitated by screening for insertion lines that exhibit reduced transmission of an associated selectable marker. Although hundreds of mutants defective in gametophyte development have been found over the years, the identities of genes responsible for these mutant phenotypes have often not been confirmed. Because large deletions and translocations are common in T-DNA and transposon insertion lines of *Arabidopsis* [36]–[39], and these chromosomal aberrations often result in lethality before fertilization [35], large datasets of gametophyte essentials that include candidate genes represented by a single mutant allele should be viewed with caution.

In order to characterize the cellular disruptions that lead to defects in embryo rather than gametophyte development, we first established a curated dataset of gametophyte essentials that could be compared with the embryo essentials found in the SeedGenes database. We then used this dataset in combination with an updated SeedGenes dataset to determine what factors most often distinguish mutants defective in embryo development from those defective in gametophyte development. Specifically, we wanted to understand how mutants defective in embryo development are able to survive gametophyte development when an essential function required throughout the life cycle is disrupted, and conversely, why some mutants exhibit gametophyte lethality instead. In addition, we sought to explain why some mutant embryos reach a later stage of development than others, and what general conclusions might be drawn about the relationship between gene function and mutant phenotype.

This analysis led us to conclude that pre-meiotic gene expression in diploid microsporocytes and megasporocytes is likely to be an important feature of reproductive development in *Arabidopsis*. Although past work has elegantly demonstrated the diversity of transcripts found in male and female gametophytes [40]–[44], the approaches pursued have not generally distinguished between transcripts produced before meiosis and those produced afterward. We propose that gene products derived from transcription of the wild-type allele in heterozygous sporocytes enable some mutant gametophytes to survive the loss of an essential gene, and that embryo lethality results when these residual products are exhausted. In *Arabidopsis*, pollen tubes travel short distances in order to reach the ovule, which increases the likelihood that male gametophytes lacking an essential gene product can survive and participate in fertilization. By contrast, equivalent gene disruptions in plants with long pollen tubes, most notably maize, are more likely to result in male gametophyte lethality.

This raises another important question: how can mutant embryos disrupted in an essential gene survive beyond the globular stage of development, given that levels of gene products stored in mutant gametophytes are insufficient to support unlimited growth? Several additional factors, including gene redundancy, residual gene function, delayed activation of specialized pathways, and diffusion of products from maternal tissues likely influence the stage of development reached by mutant embryos before seed desiccation. In addition, some gene products may perform a more limited role in an essential cellular process than others, and this can allow further development than might otherwise be expected. We conclude that the datasets of genes required for embryo and gametophyte development presented here provide a useful resource for comparative studies with other multicellular eukaryotes and valuable insights into the molecular basis of reproductive lethality in a model plant.

## Results and Discussion

### The SeedGenes dataset of *EMB* genes required for embryo development

Based on the abundance of embryo-defective mutants and the frequency of duplicate alleles in mutagenesis experiments, *Arabidopsis* appears to contain 750 to 1000 *EMB* genes located throughout the genome [28], [45]. Table S1 presents an updated, comprehensive dataset of 396 *EMB* loci in *Arabidopsis*. Many of the *EMB* gene identities revealed through work in our laboratory have not been published before. The current dataset, which excludes six atypical or problematic SeedGenes loci, likely represents about 40% saturation. This is sufficient to begin addressing the diversity of protein functions required for cell viability and embryo development. Regarding the level of certainty that each gene identified is responsible for the mutant phenotype described, we recognize two distinct categories of *EMB* genes: those labeled as confirmed, either through molecular complementation or the analysis of additional mutant alleles disrupted in the same gene, and those labeled as not confirmed, where robust genetic data alone indicate close linkage between the gene and mutant phenotype (www.seedgenes.org). Approximately 100 of the *EMB* genes in Table S1 have identities that remain to be confirmed. Based on experience with other insertion lines, we estimate that >85% of these identities are correct. Genes of unknown function remain the most problematic because protein identity cannot be used to support the conclusion that an essential process has been disrupted.

**Figure 1:**
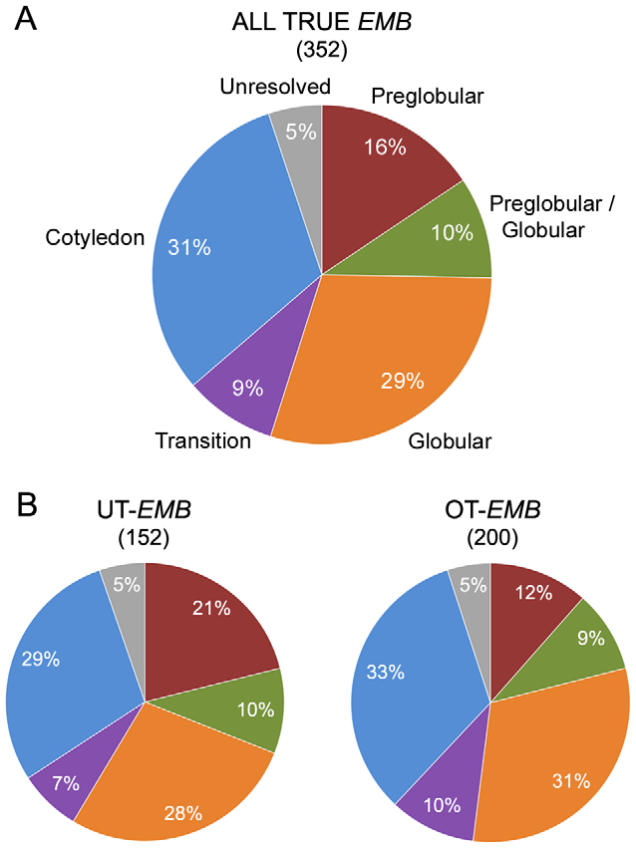
Terminal embryo phenotypes of the strongest known alleles of “true” *EMB* genes without evidence of gametophyte defects. (A) Entire dataset of 352 genes. (B) Comparison between unique “true” (UT) *EMB* genes without a redundant paralog, and the remaining dataset of other (OT) “true” *EMB* genes that may be redundant (BLASTP match>e-30).

To assess the terminal stage of development reached by mutant embryos prior to seed desiccation, we evaluated more than 700 mutants classified in SeedGenes as having an embryo-defective mutant phenotype. When more than one mutant allele was available, we chose what appeared to be the strongest allele based on phenotype and known location of the mutation. After removing 44 *EMB* loci associated with additional defects in gametophyte development, we divided the remaining 352 “true” *EMB* loci into terminal phenotype classes, ranging from preglobular to cotyledon. The results are shown in Figure 1A. Fifty-five percent of these loci are essential before the heart stage of development. Arrested embryos from these mutants are typically white, whereas many of those from mutants that reach the transition or cotyledon stages are pale green or green. More than 15% of “true” *EMB* genes are required at the earliest, preglobular stages of embryo development. Although such broad descriptions fail to capture important phenotype details, they demonstrate on a global scale that *EMB* genes differ in how far embryo development can proceed when their functions are disrupted. Furthermore, as discussed later, the existence of a substantial number of embryo-defective mutants with a Mendelian pattern of inheritance, characterized by 25% mutant seeds in siliques of selfed heterozygotes, along with a preglobular stage of embryo arrest, has implications for the timing and extent of the maternal-to-zygotic transition in gene expression during plant embryo development.

Historically, the distinction between embryo and gametophyte mutants in *Arabidopsis* has been vague and subjective. Some embryo mutants exhibit defects in gametophyte development, and some gametophyte mutants, when maintained as heterozygotes, produce a low frequency of viable mutant gametes. Embryo mutants with defects in male gametophyte development often exhibit a reduced percentage and nonrandom distribution of mutant seeds in heterozygous siliques. This “certation” effect was originally noted by Müller [46] and was later observed in collections of mutants isolated and characterized in our laboratory [27], [47]. The underlying mechanism is that disabled mutant pollen tubes often fail to reach ovules at the base of the silique. Reduced pollen viability can also result in a decreased percentage of mutant seeds overall. Embryo mutants with defects in female gametophyte development typically have a low percentage of mutant seeds, randomly distributed along the silique, combined with a high percentage of aborted ovules.

Because the SeedGenes database includes information, when available, on mutant seed ratios and distributions, we assembled a list of SeedGenes loci that appeared to have defects in both embryo and gametophyte development. These were assigned to an EMG (Embryo-Gametophyte) subclass of *EMB* loci to distinguish them from “true” *EMB* genes that lacked knockout defects in gametophyte development. We also examined publications dealing with SeedGenes loci to identify additional examples of *EMB* genes associated with defects in gametophyte development. By definition, mutants assigned to the EMG subclass are known or predicted to produce at least 10% defective seeds following self-pollination of heterozygotes and have either a reduced frequency of mutant seeds overall, too few mutant seeds at the base of the silique, or an excessive number of aborted ovules. We then established another subclass of embryo essential genes with more severe defects in gametophyte function, labeled GEM (Gametophyte-Embryo), to recognize cases where heterozygotes are known or predicted, based on reduced gamete transmission of the mutant allele, to produce between 2% and 10% mutant seeds. Both the EMG and GEM subsets of *EMB* genes, therefore, exhibit a combination of embryo and gametophyte defects. The difference lies in the extent of these defects and their impact on fertilization and embryo development. We chose 2% mutant seeds as the baseline for the GEM subclass because this corresponds to a single mutant seed per silique, on average, which is marginally above the background rate of spontaneous seed abortion [26]. By contrast, the GAM (Gametophyte) subclass of essentials represents those genes where the combined gametophyte defects are more severe, resulting in fewer than 2% mutant seeds expected from selfed heterozygotes. Different subclasses of embryo and gametophyte essentials described in this report are listed in Table 1. We then searched the literature for published examples of genes required for gametophyte development in *Arabidopsis*, recorded male and female transmission ratios when documented, and noted information about frequencies of mutant embryos produced. This provided the foundation for the curated dataset presented here of genes required for gametophyte development.

**Table 1:** Classification of genes required for embryo and gametophyte development.

Class of essential genes	Subclass	Definition of class or subclass	Genes	Table
Embryo defective		Defect in mutant embryo characterized	396	S1
	True *EMB*	No documented gametophyte defect	352	S3
	UT-*EMB*	Unique true *EMB*; No obvious paralog	152	S5
Gametophyte defective		Defect in mutant gametophyte characterized	173	S2
	GAM	<2% mutant seeds predicted or observed	70	S2
	Viable	Gametophyte defective; homozygote viable	14^a^	S2
	Uncertain	Gametophyte defective; classification uncertain	13^a^	S2
Embryo / gametophyte defective		Embryo and gametophyte defective	76	S2
	EMG	>10% mutant seeds predicted or observed	44	S2, S4
	GEM	2–10% mutant seeds predicted or observed	25	S2, S4
	GAM / GEM	Precise subclass uncertain or conflicted	4	S2
	GEM / EMG	Precise subclass uncertain or conflicted	3	S2, S4

a Included in Table S2 but not discussed in the text.

### A curated dataset of genes required for gametophyte function

In order to create a dataset of gametophyte essentials comparable to the *EMB* dataset found at SeedGenes, we excluded unconfirmed, putative gametophyte loci where only a single mutant allele was characterized and where flanking sequence was not obtained from both sides of the insert. This eliminated from consideration a sizeable number of candidate genes. But it also increased the likelihood that genes identified as being responsible for the gametophyte defects were indeed correct. The resulting dataset, shown in Table S2, contains a total of 173 genes required for normal gametophyte development. This includes 70 definitive GAM loci, 4 GAM / GEM loci, 25 GEM loci, 3 GEM / EMG loci, 44 EMG loci, and another 27 loci with defective gametophytes that either produce viable homozygotes or are difficult to classify because of incomplete transmission data. GAM loci were further differentiated based on transmission rates of the mutant allele. Three broad groups were recognized (Table S2): those with strong defects (<0.4 transmission efficiency, TE) on both the male and female sides (14 loci); those with severe defects (<0.1 TE) on the male side (44 loci); and those with severe defects (<0.1 TE) on the female side (12 loci). These 70 GAM genes defined a robust dataset of gametophyte essentials that we then compared with 352 “true” *EMB* genes devoid of gametophyte defects (Table S3) and 72 EMG and GEM loci with defects in both embryo and gametophyte development (Table S4).

### Protein functions of genes required for embryo and gametophyte development

**Figure 2:**
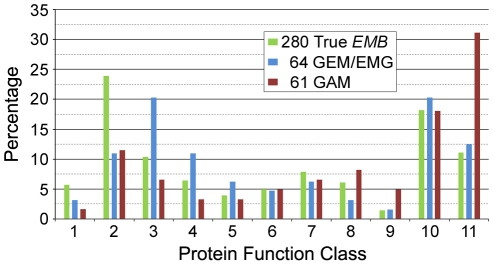
Distribution of protein function classes among different collections of essential genes. (1) DNA synthesis; repair; (2) RNA synthesis; modification; (3) protein synthesis; (4) Protein modification; transport; (5) protein degradation; (6) chromosome dynamics; (7) transcriptional regulation; (8) signaling pathways; (9) energy; electron transport; (10) metabolism; (11) cell structure; membrane function; trafficking. Significant differences are observed for function classes 2 (χ^2^ = 6.22; p<0.05), 3 (χ2 = 6.77; p<0.05), and 11 (χ^2^ = 16.7; p<0.001). Excluded are genes with functions that remain to be classified (15 “true” *EMB*; 5 EMG/GEM; 3 GAM) and those with uncertain or unknown functions (57 “true” *EMB*; 3 EMG/GEM; 6 GAM).

Extensive overlap exists between the types of protein functions required for gametophyte development and those required for embryo development (Figure 2). Consequently, one cannot explain the difference between embryo and gametophyte mutants on the basis of protein function alone. Nevertheless, distinctive features of each dataset can be identified. For example, interfering with DNA replication and RNA modification typically disrupts embryo development, whereas a complete loss of cytosolic translation in the absence of redundant gene function appears to result in 100% male and female gametophyte lethality, which means that such mutants cannot be readily maintained [48]. Blocking chloroplast translation often results in embryo arrest at the globular stage of development [49]. In fact, all of the translation defects associated with unique (non-redundant) “true” *EMB* genes identified to date involve chloroplast-localized proteins. Recently, we proposed that embryo lethality in these mutants results from a failure to produce fatty acids required for continued growth and development [49]. Interfering with translation in mitochondria typically results in an ovule abortion phenotype with features of both embryo and female gametophyte lethality, along with reduced transmission of male gametes [48]. As expected, perturbations in cellular processes required for rapid tip growth (Figure 2, Class 11) are common among mutants with severe defects in male gametophyte development. Most auxotrophic mutants exhibit embryo lethality rather than gametophyte lethality, which may be explained in part by the ability of maternal tissues to supply limited nutrients to mutant gametophytes. The disruption of male gametophyte development observed in several histidine auxotrophs placed in the EMG class of embryo lethals [50] may reflect a perturbation of the linked ATP recycling pathway rather than a loss of histidine. The abundance of EMB proteins with unknown functions included in the SeedGenes database, when compared with the gametophyte dataset, may reflect in part a reluctance of journals to publish work on individual gametophyte mutants defective in proteins with unknown functions.

### Genetic redundancy and mutant allele strength

Two potential explanations for how mutants defective in embryo development might survive gametophyte development involve genetic redundancy and mutant allele strength. The first model proposes that duplicated genes with redundant functions compensate for the loss of essential *EMB* gene functions during gametophyte development. The second model suggests that most embryo-defective mutant alleles retain some function and predicts that true null alleles of *EMB* genes should be gametophyte lethals instead. Both models are insufficient to explain the results obtained. We evaluated the level of redundancy of 352 “true” *EMB* genes using BLASTP searches (www.ncbi.nlm.nih.gov) of the *Arabidopsis* proteome. EMB proteins lacking a match with at least an e-30 level of significance were viewed as likely to be unique. Those with more significant matches were classified as potentially redundant, although in many cases these genes may still be functionally distinct. We chose an e-30 cutoff value to be relatively stringent in our selection of unique genes because we wanted to identify those *EMB* genes where survival of mutant gametophytes was least likely to result from genetic redundancy. The resulting dataset of 152 “unique true” (UT) *EMB* loci is presented in Table S5.

Distinguishing null alleles from those that retain a low level of protein function is particularly difficult with lethal mutants because homozygous mutant tissue cannot be readily analyzed. We therefore decided to record the insert locations for mutant alleles represented in the SeedGenes database, with the assumption that on average, disruptions in the beginning (A region) and middle (B region) of a gene may be more likely to eliminate protein function than those at the end of a gene (C region). We also distinguished insertions found in introns from those located in exons, although when a single flanking sequence was available and localized to an intron, we were uncertain whether an adjacent exon might also be disrupted. Overall, 89 of the 152 UT-*EMB* loci were associated with one or more mutant alleles that contained an exon insertion in the first or middle third of the gene. Another 23 had an intron insertion in the same (A/B) region, and 19 contained an insertion in the final third of the gene. When we compared the terminal phenotypes of 89 mutants carrying A/B exon disruptions with those of 42 mutants containing A/B intron or C region disruptions, we found no significant differences (χ^2^ = 2.50; p = 0.65) in the frequencies of early versus late embryo phenotypes (data not shown). We also found no significant differences (χ^2^ = 7.69; p = 0.17) in the phenotype distributions obtained when the unique and potentially redundant datasets of “true” *EMB* genes were compared (Figure 1B), although mutants with preglobular defects appeared to be somewhat more common among UT-*EMB* genes, consistent with the model that some mutants with late defects may be partially rescued by expression of a second, redundant gene.

Another point that argues against the idea that most embryo-defective mutants correspond to weak alleles of genes required for gametophyte development is that relatively few examples of gametophyte lethal alleles of known *EMB* loci have been documented in the literature. We have uncovered several cases, including *EMB1989/NRPB2*, *EMB2779/LCB1*, *RSW1/CESA1*, *EMB24*/*AtASP38*, and *EMB2776/PRP4/LIS* [51]–[54], but the number is small when compared with the total number of essential genes identified to date. By contrast, more than 30 examples have been documented of weak mutant alleles of *EMB* genes that complete embryo development and exhibit other defects as seedlings or adult plants (Table S6). Based on these analyses, we conclude that in the vast majority of cases, the ability of *emb* mutant gametophytes to participate in fertilization is not likely to be explained by residual protein function provided by a weak mutant allele. Other factors besides genetic redundancy and weak mutant alleles must, therefore, be involved in supporting gametophyte development in heterozygous plants.

### Significance of terminal embryo phenotypes

Disruptions of some UT-*EMB* genes permit growth and development to continue beyond the globular stage of embryo development even though the mutant alleles in question appear to be nulls. How can this apparent contradiction be explained? Because diploid sporocytes are small in comparison to multicellular embryos late in development, they are unlikely to provide gametophytes with sufficient quantities of stored gene products to explain the late embryo phenotypes found in these mutants. Other factors must therefore be involved. In order to evaluate such loci, we focused on 36 UT-*EMB* genes with A/B exon disruptions that exhibited a terminal embryo phenotype at the transition or cotyledon stage. One striking feature of the resulting dataset, shown in Table S7, is the diversity of possible causes for the late embryo phenotypes observed. These include diffusion of an essential nutrient from surrounding maternal tissues (*bio1*), late expression of a transcriptional regulator (*wox2*), delayed requirements for specialized pathways in metabolism (*tag1*, *hyd1*, *emb1873*), and peripheral roles of EMB proteins in essential cellular processes (*pex10*, *emb2184*, *emb2754*). Other notable features of this dataset include the abundance of genes encoding chloroplast-localized proteins (15 loci) and proteins with unknown cellular functions (12 loci). We conclude that in some cases, the late embryo phenotypes observed in these knockouts are consistent with known protein functions that become important late in embryo development. In other words, elimination of these specialized functions would not be expected to result in lethality immediately after fertilization. In other cases, the protein product likely performs a peripheral role in an essential process required throughout growth and development. The survival of mutant gametophytes in *emb/EMB* heterozygotes is best addressed, therefore, using *EMB* genes with knockout phenotypes at early stages of embryo development, because in those cases, survival of the mutant gametophyte contrasts with the documented, early lethality found in the mutant embryo.

We then focused on a dataset of 25 UT-*EMB* genes with a preglobular or zygotic terminal phenotype, which is more consistent with an essential gene product required throughout development (Table 2). Once again, a wide range of protein functions is represented, from purine, proline, and pantothenate biosynthesis (*fac1, pts, emb2772*) and tRNA modification in the cytosol (*emb2191, emb2820*) to chromosome dynamics (*emb2773, emb2782*) and microtubule assembly (*ttn1, pfi, por, emb2804, qqt2*). Surprisingly, chloroplast-localized proteins are missing from this dataset and genes encoding proteins with unknown functions are reduced in occurrence. We propose that in some of these knockouts, residual gene products from the wild-type allele transcribed in diploid sporocytes enable mutant gametophytes to participate in fertilization. Development of homozygous mutant seeds arrests a short time later, when these reserves become depleted. Diffusion of nutrients from surrounding maternal tissues cannot be the primary source of metabolites for all stages of male gametophyte development because pollen-tube growth in *Arabidopsis* can proceed *in vitro* on a simple, defined medium [44], [55].

**Table 2:** Unique “true” *EMB* genes with a documented preglobular embryo phenotype.

Locus	Gene symbol	Identity status^a^	Function class^b^	Predicted protein function
At1g03360	*RRP4*	C	2.3	rRNA Processing; Exonuclease
At1g09770	*AtCDC5*	NC	7	MYB Domain Transcription Factor; Cell Cycle Control
At1g48175	*EMB2191*	C	2.3	tRNA Adenosine Deaminase
At1g55350	*EMB1275*	C	12	Calpain-Type Cysteine Protease
At1g55900	*EMB1860*	C	4.2	Inner Mitochondrial Membrane Import Protein
At1g71440	*PFI*	C	11.1	Tubulin Folding Cofactor E; Microtubule Dynamics
At2g30920	*EMB3002*	NC	9	Methyltransferase; Ubiquinone Biosynthesis
At2g32590	*EMB2795*	C	6.2	Condensin Complex Subunit; Sister Chromatid Segregation
At2g34780	*EMB1611*	C	13	Uncertain
At2g38280	*FAC1*	C	10.1	AMP Deaminase; Purine Biosynthesis
At2g38670	*PECT1*	C	10.5	Phosphatidylethanolamine Biosynthesis
At3g10220	*EMB2804*	C	11.1	Tubulin Folding Cofactor B; Microtubule Dynamics
At3g20070	*TTN9*	C	13	Unknown
At3g60740	*TTN1*	C	11.1	Tubulin Folding Cofactor D; Microtubule Dynamics
At4g03240	*AtFH*	C	12	Frataxin; Mitochondrial Fe-S Protein Biogenesis
At4g21800	*QQT2*	C	11.1	ATP/GTP Binding Protein; Microtubule Localization
At4g39920	*POR*	C	11.1	Tubulin Folding Cofactor C; Microtubule Dynamics
At5g13480	*FY*	C	2.3	Nuclear RNA Binding / Polyadenylation Protein
At5g13690	*CYL1*	C	10.4	Alpha-N-Acetyl-Glucosaminidase
At5g14800	*EMB2772*	C	10.1	Proline Biosynthesis
At5g15540	*EMB2773*	C	6.2	Adherin; Sister Chromatid Cohesion
At5g15920	*EMB2782*	C	6.2	SMC Family Protein; Chromosome Dynamics
At5g24670	*EMB2820*	C	2.3	tRNA Adenosine Deaminase
At5g48840	*PTS*	C	10.1	Pantothenate Synthetase
At5g59440	*ZEUS1*	C	10.1	Thymidylate Kinase; Nucleotide Biosynthesis

a Gene responsible for mutant phenotype confirmed (C) or not confirmed (NC) through allelism tests or molecular complementation.

b Protein function classes are defined in Table S1, last spreadsheet tab.

### Nature of stored gene products

In principle, the gene products contributed by diploid sporocytes to mutant gametophytes could be either RNA or protein, or a combination of both. Male gametophytes have long been known to be active in transcription. Post-meiotic gene expression was first demonstrated in maize by analyzing isozyme patterns for dimeric enzymes in pollen from heterozygous plants [56]. Female gametophytes have been more difficult to study, due in part to the presence of surrounding maternal tissues. Translation of cytosolic mRNAs is an essential feature of gametophyte development in *Arabidopsis*. Mutant alleles unable to support this essential process are likely not transmitted through male or female gametes [48]. Furthermore, pollen-tube growth *in vitro* is blocked in the presence of translation inhibitors [42]. This means that diploid sporocytes do not contribute to haploid gametophytes sufficient levels of functional proteins derived from all essential genes required to complete growth and development. Nevertheless, the possibility remains that stored products of some essential genes might be proteins instead of RNAs.

Knockout mutants disrupted in essential subunits of RNA polymerase are unable to transmit the mutant allele through female gametes and exhibit reduced transmission of the mutant allele through male gametes [51]. On the female side, this indicates that transcription within the gametophyte is required for normal development and that maternal sources of RNA polymerase are insufficient to meet transcriptional demands. On the male side, microsporocytes appear to contribute RNA polymerase activity to male gametophytes, either as stored transcript or functional protein, but this activity is not sufficient to meet the full transcriptional demand in mutant pollen, resulting in reduced rates of transmission. RNA polymerase knockouts, however, fail to address the broader question of which other products of essential genes are contributed from sporocytes to gametophytes, and whether some of those products are proteins rather than transcripts. A different pattern has been documented for the *SHORT SUSPENSOR* (*SSP*) locus, where a transcript produced in male gametophytes is translated in the zygote and modulates an important signaling pathway early in embryo development [57].

### Transcriptional profiles of essential genes in male gametophytes

We searched for evidence of stored, pre-meiotic transcripts of *EMB* genes in haploid gametophytes by comparing the published transcript profiles [42]–[45] for several different classes of essential genes in *Arabidopsis*: (a) 59 GAM or GEM loci with severe defects in male transmission (Table S8); (b) 48 GEM or EMG loci with moderate defects in male transmission (Table S9); and (c) 75 UT-*EMB* loci with functional male gametophytes and a preglobular (25 loci; Table 2) or globular (50 loci; Table S10) stage of embryo arrest. We expected to find higher levels of transcript in young microspores than in pollen tubes or mature pollen grains when pre-meiotic transcripts supported male gametophyte development. By contrast, we anticipated that transcript levels would increase or remain steady throughout gametophyte development for GAM loci with severe defects in male transmission, consistent with post-meiotic gene expression. EMG and GEM loci with a combination of embryo defects and moderate defects in male gametophyte development were expected to have intermediate or variable profiles.

Our analysis of male transcriptome datasets is summarized in Table 3. Overall, the results from different laboratories are remarkably consistent and highlight informative trends. Transcripts from UT- *EMB* loci with early defects in seed development are more likely to be absent from mature pollen (χ^2^ = 17.1; p<0.001) than transcripts from gametophyte loci with severe defects in male transmission. This suggests that survival of *emb* mutant pollen tubes often depends on proteins synthesized during pollen development and not on stored mRNAs translated after pollen maturation. This model predicts that EMB proteins should be present in mature pollen grains when their transcripts are absent. Unfortunately, the published proteome datasets for *Arabidopsis* [58]–[62] are too limited in scope to support or refute this prediction. Only a handful of proteins derived from UT-*EMB* genes with early stages of embryo arrest are included in these datasets, and even the GAM, GEM, and EMG classes are infrequently represented (Table S11).

**Table 3:** Summary of pollen development transcriptome data for selected essential genes^a^.

	Unique true *EMB* genes^b^		Male gametophyte defectives^c^		
Transcript accumulation pattern found in male gametophytes	Preglobular^d^	Globular^d^	Moderate^d^	Severe^d^	Reference
Transcript detected early in pollen development but not in mature pollen	70%	64%	55%	27%	[42]
Transcript detected throughout pollen development	17%	29%	43%	61%	[42]
Other patterns of transcript accumulation	13%	7%	2%	12%	[42]
Transcript detected throughout pollen development; ≥2× higher at late stages	0%	2%	2%	26%	[42]
Transcript detected throughout pollen development; ≥2× higher at early stages	4%	10%	12%	13%	[42]
**Comparison of results obtained from different laboratories**					
Transcript detected in mature pollen^e^	26%	36%	43%	69%	[42]
Substantial transcript detected in mature pollen^f^^,^ g	9%	12%	21%	47%	[42]
Transcript detected in mature pollen^e^^,^ g	13%	14%	29%	42%	[43]
Transcript detected in mature pollen^g^	13%	19%	21%	51%	[44]
Transcript detected in pollen tube^g^	13%	14%	21%	45%	[44]

a Based on published, large-scale microarray datasets for wild-type plants [42]–[44].

b Non-redundant genes with embryo arrest stage shown; no gametophyte defects observed.

c Moderate (EMG and GEM) and severe (GAM) classes are described in the text.

d Percentages of essential genes with transcript detected are noted. Total number of genes analyzed: preglobular (23/25); globular (42/50); moderate (42/48); severe (51/59). Excluded genes were not part of the microarray dataset or had no transcript detected during pollen development.

e Level detected was among top 50% of all transcript levels recorded.

f Level detected was among top 25% of all transcript levels recorded. This cutoff value for detection [42] gave results more consistent with those obtained elsewhere [43], [44].

g Most of the genes identified with a positive signal using data from one laboratory were the same as those identified using data from the other laboratories.

Transcripts from UT-*EMB* genes with early embryo defects are frequently detected in young microspores, and their levels do not increase substantially during pollen development. Both the transcript profiles and inheritance patterns of these *EMB* genes are therefore consistent with the utilization of stored transcript during pollen development and inconsistent with gametophyte expression. By contrast, genes required for male gametophyte development are often up-regulated during pollen development and their transcripts are frequently detected both at pollen maturity and in pollen tubes. As expected, EMG and GEM loci with moderate defects in male transmission have transcript profiles intermediate between those of preglobular *EMB* and severe male gametophyte loci. Transcript profiles for *EMB* genes with a globular stage of arrest are more difficult to interpret but remain consistent with pre-meiotic transcription and inconsistent with widespread storage of transcripts in mature pollen. Globular mutant embryos do not appear to arrest later in development simply because their *EMB* transcripts are stored at higher levels in mature pollen. Whether the protein products of these transcripts are more abundant in pollen and are retained in sufficient levels after fertilization to support further embryo development remains an open question.

### Transcriptional profiles of essential genes in microsporocytes

To test our prediction that transcripts of *EMB* genes are often contributed from diploid microsporocytes to haploid gametophytes, we evaluated three recent reports of transcript profiles for isolated male meiocytes. Two of these studies utilized direct RNA sequencing [63], [64]; the other relied on microarray analysis [65]. One report [64] characterized the full transcriptional landscape of isolated microsporocytes; the other two [63], [65] emphasized the identification of meiosis-specific gene products. Of the 183 GAM, GEM, EMG, and *EMB* genes listed in Table 2 and Tables S8, S9, S10 combined, just one is included among the 844 meiosis-specific loci described by Yang et al. [64]. This is not surprising because most of the genes in our dataset encode proteins thought to be required at multiple stages of development. Similarly, only 5% of the 183 essential genes in our dataset are included among 752 transcripts that Libeau et al. [65] conclude are enriched in microsporocytes when compared with leaves and roots. This is intriguing because it suggests that microsporocytes do not amplify essential gene transcripts in preparation for subsequent use during male gametophyte development.

This model is further supported by our analysis of the dataset of Yang et al. [64], which is shown in Table 4. As expected, transcripts from about 85% of UT-*EMB* genes with early embryo defects are detected in microsporocytes, compared with just 55% for all protein-coding genes. Many of these transcripts should remain functional in young microspores. Surprisingly, transcripts from a similar percentage of GAM or GEM loci with defects in male transmission are also detected in microsporocytes. This contrasts with the known inheritance pattern of gametophyte defects observed. Furthermore, the frequency of genes with abundant transcripts (>15.0 RPKM) is not significantly higher for UT-*EMB* genes with early embryo defects than for male gametophyte essentials (χ^2^ = 0.61; p = 0.44), or for all genes with transcripts detected in microsporocytes (χ^2^ = 0.22; p = 0.64). We therefore propose that in most cases where transcription of an *EMB* gene in microsporocytes supports gametophyte development, survival of mutant (*emb*) gametophytes results from relatively modest levels of residual transcript contributed to young microspores and subsequently translated during pollen development. By contrast, male gametophyte defects in gametophyte lethals arise when post-meiotic expression of an essential gene is required to supplement whatever functional transcript remains from pre-meiotic expression in microsporocytes. Confirmation of this model could be achieved in the future by determining whether down-regulation of an *EMB* gene in microsporocytes changes an embryo lethal into a gametophyte lethal.

**Table 4:** Summary of microsporocyte transcriptome data for selected essential genes^a^.

	Unique true *EMB* genes^b^		Male gametophyte defectives^c^		
Transcript detected in microsporocytes^d^	Preglobular	Globular	Moderate	Severe	Microsporocyte dataset in full
≥1.0 RPKM	88%	82%	90%	81%	55%
>5.0 RPKM^e^	41%	41%	53%	52%	49%
>15.0 RPKM^e^	14%	17%	23%	19%	18%

a Based on published transcriptome dataset of Yang et al. [64]. Percentages of essential genes with transcripts detected at different abundance levels are noted.

b Non-redundant genes with early embryo arrest and no gametophyte defects. Genes analyzed: preglobular [25]; globular [50].

c Moderate (EMG and GEM) and severe (GAM) classes are described in the text. Genes analyzed: moderate [48]; severe [59].

d Reads per kilobase per million reads (RPKM); high numbers indicate abundant transcripts.

Transcripts with RPKM≥1.0 considered present.

e Genes with no transcript detected and those with RPKM<1.0 are excluded from the percentages shown.

### Transcriptional profiles of essential genes in megasporocytes

We focused our analysis on genes required for male gametophyte development because data for female meiocytes are more difficult to obtain and evaluate. One could argue that because the number of confirmed, female gametophyte mutants identified to date is fewer than the number of male gametophyte mutants, megasporocytes may be more effective than microsporocytes at contributing functional gene products to progeny haploid cells. This cannot, however, be true for all essential genes, because RNA polymerase knockouts are not transmitted through the female side, which indicates that some essential genes must be transcribed in female gametophytes. While it seems logical that knockouts of genes required for rapid tip growth should exhibit male rather than female gametophyte lethality, the mechanism responsible for severe female defects in knockouts with minimal disruption of male gametophytes remains obscure. In some cases, gene duplication may be involved, with a redundant gene supplying the missing function on the male side when the paralog required for female development is disrupted.

The recent publication of transcriptional profiles for *Arabidopsis* megasporocytes [66] and female gametophytes [67] makes it possible to analyze maternal contributions to haploid gametophytes and the role of pre-meiotic transcription in supporting embryo development. Transcripts of many essential genes are detected in megasporocytes. Of the 75 UT-*EMB* genes with early embryo defects, 62% produced transcripts detected in at least three of four megasporocyte samples analyzed [66]. This is consistent with the model that some transcripts produced in megasporocytes are used to support female gametophyte development. We then asked whether genes associated with severe defects in female transmission often fail to produce transcripts detected in megasporocytes. Unexpectedly, when 19 such genes (Table S2) were examined, the likelihood of transcripts being present in the megasporocyte dataset [66] was not significantly different (x^2^ = 0.24; p = 0.62) from that observed for the 75 UT-*EMB* genes with early phenotypes, whose knockouts survive gametophyte development. This result mirrors the trend noted above for microsporocyte transcripts involving male gametophyte essential genes. Furthermore, we found no significant difference (χ^2^ = 1.81; p = 0.40) in the likelihood of transcripts being present in megasporocytes, and available in the egg cell for contribution to the zygote, when we compared our datasets of UT-*EMB* genes with preglobular, globular, and cotyledon mutant phenotypes. We have therefore been unable to establish a role for pre-meiotic transcripts in supporting the continued growth of mutant embryos to a globular stage and beyond. By contrast, observed differences in the severity of phenotypes between male and female gametophyte mutants disrupted in essential genes, and between embryo and gametophyte lethals overall, appear in some cases to reflect differences in the amount of residual, essential gene products donated from sporocytes to developing gametophytes. The practical applications of these different patterns of reproductive lethality in *Arabidopsis* remain to be explored.

### The transition from maternal to zygotic gene expression

In addition to evaluating sporocyte transcription and gametophyte viability in embryo-defective mutants of *Arabidopsis*, we utilized the dataset of *EMB* genes presented here to address another central issue in plant development related to stored, essential gene products. Two fundamental questions have figured prominently in recent discussions of gene expression during plant embryo development. One involves the extent of paternal allele contributions to the developing embryo and endosperm tissue. The other concerns when during development the transition from maternal to zygotic gene expression takes place. Both of these questions have been widely studied in maize [68]–[70] and *Arabidopsis* [71]–[77]. However, significant conflicts remain over the interpretation of results obtained, reliability of methods employed, and validity of conclusions drawn.

Our interest in this field began almost 20 years ago, when we isolated and characterized a mutant (*emb173*) with an unusual pattern of inheritance: heterozygous plants produced 50% mutant seeds regardless of pollen genotype [36]. Additional alleles of this gene, later named *MEA*, were isolated and characterized elsewhere, the gene was shown to encode a polycomb group protein that regulates cell proliferation, and the paternal allele was found to be imprinted by DNA methylation, particularly in the endosperm [78]–[80]. Several other genes with a similar pattern of inheritance [www.seedgenes.org] but differing seed phenotypes and mechanisms of maternal influence have also been described [81], [82]. Evaluation of these genes and mutant phenotypes has frequently centered on maternal genome contributions and paternal genomic imprinting in relation to parental conflict over resource allocation from mother to offspring. Despite considerable interest in the origin and significance of these loci, their numbers are quite limited when compared to *EMB* genes with a normal, zygotic pattern of inheritance.

We were therefore surprised by a report 12 years ago that claimed widespread, delayed activation of the paternal genome during seed development in *Arabidopsis* and concluded that early embryo and endosperm development are primarily, if not exclusively, under maternal control [71]. This conclusion, which appeared inconsistent with the inheritance pattern of early embryo-lethal mutants, was based on the analysis of ß-glucuronidase (GUS) enhancer trap lines that marked the expression of 19 different genes known to be active during ovule and early seed development. By comparing the presence or absence of GUS staining in embryos produced through reciprocal crosses to wild-type plants, the authors concluded that the paternal gene in each case was not expressed until 3 to 4 days after pollination, when the embryo had reached the early globular stage. Additional studies with *EMB30/GNOM* and tests for allele-specific single nucleotide polymorphisms (SNPs) in transcripts produced following reciprocal crosses between accessions seemed to support these observations. This model was soon challenged through further work on *Arabidopsis* [72]. With maize, published reports were both consistent [68] and inconsistent [69] with the model. Down-regulating RNA polymerase II activity after fertilization was later found to interfere with endosperm but not early embryo development in *Arabidopsis* [73]. Once again, this suggested that the embryo is transcriptionally quiescent before the globular stage and is dependent instead on stored maternal transcripts.

Recently, a large-scale RNA sequencing effort [77] designed to detect accession-specific SNPs from *Arabidopsis* embryos at the 2–4 cell stage indicated that genome-wide, transcripts from the maternal allele predominate over those from the paternal allele through at least the globular stage of development. Following a cross between Columbia and Landsberg *erecta* accessions, almost 90% of the 135,000 informative sequence reads derived from 4,000 loci genome-wide appeared to be of maternal origin at the 2–4 cell embryo stage. Overall, the paternal contribution increased somewhat by the globular stage, and some loci produced equal amounts of transcripts from both maternal and paternal alleles, but early in development, the vast majority of loci seemed to be under maternal control. The authors stated that their work reconciled observations made by different laboratories of both maternal and zygotic effects during embryogenesis in *Arabidopsis*. But their data still conflict with the existence of large numbers of embryo-defective mutants with a Mendelian pattern of inheritance and cellular defects observed well before the globular stage.

Because all of the publications on the maternal-to-zygotic transition in *Arabidopsis* have cited at most a handful of *EMB* genes with known defects early in development, we decided to establish a more robust dataset of known *EMB* genes with arrested embryos that fail to reach the globular stage. The resulting collection of 70 genes, corresponding to about 18% of all 396 *EMB* genes identified to date, is presented in Table S12. This dataset does not include genes such as *EMB30/GNOM*, where the initial defects are observed long before embryo development becomes arrested, and other mutants with late terminal phenotypes where early defects remain to be uncovered. Nevertheless, with this dataset in hand, we were intrigued to learn of another study on the maternal-to-zygotic transition in *Arabidopsis* that followed an approach similar to Autran *et al.* [77] but expanded the number of genes and embryo stages examined, included the analysis of reciprocal crosses, and increased the washing of isolated embryos to remove contaminating RNA derived from the maternal seed coat (Michael Nodine and David Bartel, personal communication). Their results for more than 7,000 loci present a different picture, with maternal and paternal transcripts for most genes found in equal amounts, even at the 1–2 cell stage. Transcripts from the 70 preglobular *EMB* genes listed in Table S12 exhibit a similar, bi-parental pattern of accumulation, consistent with their known mode of inheritance.

When these results are evaluated in the context of several decades of research on embryo-defective mutants, we believe there is compelling evidence for early activation of the zygotic genome in *Arabidopsis*, and that unlike the situation observed in most animal systems, early expression of essential genes is required to support a wide range of cellular functions beginning shortly after fertilization. From a historical perspective, elucidating the role of paternal gene expression in plant embryo development provides a compelling illustration of the value of large datasets of genes with essential functions during growth and development. A more detailed picture of the molecular basis of reproductive lethality in *Arabidopsis* should emerge as additional genes with essential functions are identified in the future.

## Materials and Methods

### Updating the published dataset of *EMB* genes in *Arabidopsis*

The last published, comprehensive dataset of *EMB* genes [30] was compiled more than 7 years ago and included a total of 220 loci, 70% of which were uncovered through a forward genetic screen undertaken in collaboration with Syngenta [29]. Since that time, the SeedGenes database has undergone two major updates to include many additional genes and mutant alleles. In addition to curating information from the literature, several large-scale, reverse genetic approaches were pursued in our laboratory to identify *EMB* genes that were missed in past screens [24]. These included the analysis of candidate genes encoding proteins that shared a common cellular process (aminoacylation of tRNAs), metabolic pathway (biotin and histidine biosynthesis), and intracellular compartment (chloroplast) with products of known *EMB* genes [48]–[50], [83]. We also used PCR (polymerase chain reaction) to amplify plant sequences flanking T-DNA inserts for several mutants that had been mapped but not cloned [45]. In addition, we analyzed candidate genes that represented potential homologs of essential genes identified in other organisms [30], genes that encoded proteins thought to interact with a known EMB protein, and hundreds of insertion lines corresponding to genes for which the Ecker project on providing knockouts for every gene [38] failed to identify viable homozygotes. Whenever possible, genetic crosses between heterozygotes were used to confirm allelism and demonstrate that the gene responsible for the mutant phenotype had been identified. Crosses were also used to identify new alleles of known *EMB* genes that had previously been mapped but not cloned [45]. Methods used in our laboratory to analyze embryo-defective mutants are presented at the tutorial section of SeedGenes and in several recent publications [24], [45], [48]–[50], [83]. Although genetic and phenotypic data on the most promising mutants have consistently been added to the SeedGenes database, some of the *EMB* genes identified in our laboratory and found in Table S1 are published here for the first time.

### Establishing a dataset of genes required for gametophyte development

Several approaches were taken to identify and evaluate genes reported to be required for gametophyte development in *Arabidopsis*. We began with a list of all known genes with a mutant phenotype published 8 years ago [84], examined candidate genes from The Arabidopsis Information Resource (www.arabidopsis.org) that were thought to be associated with phenotype information, and performed a variety of PubMed searches (www.ncbi.nlm.nih.gov) with relevant keywords (gamete, gametophyte, mutant, mutation, knockout, lethal, essential, male, female, and pollen). Publications describing the large-scale identification of genes required for male and female gametophyte development [31], [35] were evaluated to identify genes most likely to be responsible for the gametophyte defects observed. Emphasis was placed on genes with multiple mutant alleles and flanking sequence derived from both sides of the insert. We also utilized information presented at SeedGenes and in publications on embryo-defective mutants to determine which mutants exhibited additional defects in gametophyte development. Transmission efficiency (TE) of the mutant allele, determined in crosses between heterozygous and wild-type plants, was defined as: (number of heterozygous F_1_ plants) / (number of wild-type F_1_ plants). Subclasses of genes with different mixtures of embryo and gametophyte defects (GAM, GEM, EMG) and levels of transmission reduction (e.g. 0 ♂; MMM, MM, M, +) are defined in the second and third tabbed spreadsheets in Table S2. The percentage of mutant seeds expected in selfed siliques of heterozygotes with reduced transmission of the mutant allele through one or both gametes was calculated as: [(♂ TE)/(1+♂ TE )]×[(♀ TE)/(1+♀ TE)]×100, where “1” represents normal transmission efficiency of the wild-type allele. This reduces to [1/(1+1)]×[1/(1+1)]×100, or 25% mutant seeds in the absence of defects in gamete transmission.

## Supporting Information

Table S1 **Updated SeedGenes dataset of 396** ***EMB*** **genes of** ***Arabidopsis*** **.**
(XLS)

Table S2 **173** ***Arabidopsis*** **genes with a gametophyte-defective phenotype.**
(XLS)

Table S3 **352 true** ***EMB*** **genes without evidence of gametophyte defects.**
(XLS)

Table S4 **72 GEM and EMG genes with both embryo and gametophyte defects.**
(XLS)

Table S5 **152 unique true** ***EMB*** **genes of** ***Arabidopsis*** **.**
(XLS)

Table S6 **Examples of** ***EMB*** **genes with other phenotypes in weak alleles or RNAi lines.**
(XLS)

Table S7 **36 unique true** ***EMB*** **genes with late phenotypes in putative knockout alleles.**
(XLS)

Table S8 **59 GAM or GEM genes with severe defects in male gamete transmission.**
(XLS)

Table S9 **48 GEM or EMG genes with moderate defects in male gamete transmission.**
(XLS)

Table S10 **50 unique true** ***EMB*** **genes with early globular to globular phenotypes.**
(XLS)

Table S11 **Detection of proteins required for male gametophyte and embryo development in pollen.**
(XLS)

Table S12 **70** ***EMB*** **genes where mutant embryos arrest at the preglobular stage of development.**
(XLS)

## References

[1] Osterman AL, Gerdes SY (2008). Microbial Gene Essentiality: Protocols and Bioinformatics. Methods in Molecular Biology (Vol 416).

[2] Msadek T (2009). Grasping at shadows: Revealing the elusive nature of essential genes.. J Bacteriol.

[3] Ji Y, Zhang B, Van Horn SF, Warren P, Woodnutt G (2001). Identification of critical *Staphylococcal* genes using conditional phenotypes generated by antisense RNA.. Science.

[4] Akerley BJ, Rubin EJ, Novick VL, Amaya K, Judson N (2002). A genome-scale analysis for identification of genes required for growth or survival of *Haemophilus influenzae*.. Proc Natl Acad Sci USA.

[5] Kobayashi K, Ehrlich SD, Albertini A, Amati G, Andersen KK (2003). Essential *Bacillus subtilis* genes.. Proc Natl Acad Sci USA.

[6] Baba T, Ara T, Hasegawa M, Takai Y, Okumura Y (2006). Construction of *Escherichia coli* K-12 in-frame, single-gene knockout mutants: The Keio collection.. Mol Syst Biol.

[7] Giaever G, Chu AM, Ni L, Connelly C, Riles L (2002). Functional profiling of the *Saccharomyces cerevisiae* genome.. Nature.

[8] Ben-Aroya S, Coombes C, Kwok T, O'Donnell KA, Boeke JD (2008). Toward a comprehensive temperature-sensitive mutant repository of the essential genes of *Saccharomyces cerevisiae*.. Mol Cell.

[9] Hillenmeyer ME, Fung E, Wildenhain J, Pierce SE, Hoon S (2008). The chemical genomic portrait of yeast: Uncovering a phenotype for all genes.. Science.

[10] Kim DU, Hayles J, Kim D, Wood V, Park HO (2010). Analysis of a genome-wide set of gene deletions in the fission yeast *Schizosaccharomyces pombe*.. Nat Biotechnol.

[11] Zhang R, Lin Y (2009). DEG 5.0, a database of essential genes in both prokaryotes and eukaryotes.. Nucleic Acids Res.

[12] Perrimon N, Lanjuin A, Arnold C, Noll E (1996). Zygotic lethal mutations with maternal effect phenotypes in *Drosophila melanogaster*. II. Loci on the second and third chromosomes identified by P-element-induced mutations.. Genetics.

[13] Boutros M, Kiger AA, Armknecht S, Kerr K, Hild M (2004). Genome-wide RNAi analysis of growth and viability in *Drosophila* cells.. Science.

[14] Dietzl G, Chen D, Schnorrer F, Su KC, Barinova Y (2007). A genome-wide transgenic RNAi library for conditional gene inactivation in *Drosophila*.. Nature.

[15] Kamath RS, Fraser AG, Dong Y, Poulin G, Durbin R (2003). Systematic functional analysis of the *Caenorhabditis elegans* genome using RNAi.. Nature.

[16] Mouse Genome Informatics (MGI) Website.. http://www.informatics.jax.org.

[17] Brown SDM, Wurst W, Kühn R, Hancock JM (2009). The functional annotation of mammalian genomes: The challenge of phenotyping.. Annu Rev Genet.

[18] Silva JM, Marran K, Parker JS, Silva J, Golding M (2008). Profiling essential genes in human mammary cells by multiplex RNAi screening.. Science.

[19] Park D, Park J, Park SG, Park T, Choi SS (2008). Analysis of human disease genes in the context of gene essentiality.. Genomics.

[20] Liang H, Li WH (2009). Functional compensation by duplicated genes in mouse.. Trends Genet.

[21] Chen S, Zhang YE, Long M (2010). New genes in *Drosophila* quickly become essential.. Science.

[22] Liao BY, Zhang J (2008). Null mutations in human and mouse orthologs frequently result in different phenotypes.. Proc Natl Acad Sci USA.

[23] Liao BY, Weng MP, Zhang J (2010). Contrasting genetic paths to morphological and physiological evolution.. Proc Natl Acad Sci USA.

[24] Meinke D, Muralla R, Sweeney C, Dickerman A (2008). Identifying essential genes in *Arabidopsis thaliana*.. Trends Plant Sci.

[25] Meinke D, Muralla R, Dickerman A (2009). An essential line of inquiry.. Trends Plant Sci.

[26] Meinke DW, Sussex IM (1979). Embryo-lethal mutants of *Arabidopsis thaliana*: A model system for genetic analysis of plant embryo development.. Devel Biol.

[27] Meinke DW (1985). Embryo-lethal mutants of *Arabidopsis thaliana*: Analysis of mutants with a wide range of lethal phases.. Theor Appl Genet.

[28] Franzmann LH, Yoon ES, Meinke DW (1995). Saturating the genetic map of *Arabidopsis thaliana* with embryonic mutations.. Plant J.

[29] McElver J, Tzafrir I, Aux G, Rogers R, Ashby C (2001). Insertional mutagenesis of genes required for seed development in *Arabidopsis thaliana*.. Genetics.

[30] Tzafrir I, Pena-Muralla R, Dickerman A, Berg M, Rogers R (2004). Identification of genes required for embryo development in *Arabidopsis*.. Plant Physiol.

[31] Pagnussat GC, Yu HJ, Ngo QA, Rajani S, Mayalagu S (2005). Genetic and molecular identification of genes required for female gametophyte development and function in *Arabidopsis*.. Development.

[32] Procissi A, de Laissardière S, Férault M, Vezon D, Pelletier G (2001). Five gametophytic mutations affecting pollen development and pollen tube growth in *Arabidopsis thaliana*.. Genetics.

[33] Lalanne E, Michaelidis C, Moore JM, Gagliano W, Johnson A (2004). Analysis of transposon insertion mutants highlights the diversity of mechanisms underlying male progamic development in *Arabidopsis*.. Genetics.

[34] Johnson MA, von Besser K, Zhou Q, Smith E, Aux G (2004). Arabidopsis *hapless* mutations define essential gametophytic functions.. Genetics.

[35] Boavida LC, Shuai B, Yu HJ, Pagnussat GC, Sundaresan V (2009). A collection of Ds insertional mutants associated with defects in male gametophyte development and function in *Arabidopsis thaliana*.. Genetics.

[36] Castle LA, Errampalli D, Atherton TL, Franzmann LH, Yoon ES (1993). Genetic and molecular characterization of embryonic mutants identified following seed transformation in *Arabidopsis*.. Mol Gen Genet.

[37] Tax FE, Vernon DM (2001). T-DNA-associated duplication/translocations in *Arabidopsis*. Implications for mutant analysis and functional genomics.. Plant Physiol.

[38] O'Malley RC, Ecker JR (2010). Linking genotype to phenotype using the *Arabidopsis* unimutant collection.. Plant J.

[39] Clark KA, Krysan PJ (2010). Chromosomal translocations are a common phenomenon in *Arabidopsis thaliana* T-DNA insertion lines.. Plant J.

[40] Johnston AJ, Meier P, Gheyselinck J, Wuest SE, Federer M (2007). Genetic subtraction profiling identifies genes essential for *Arabidopsis* reproduction and reveals interaction between the female gametophyte and the maternal sporophyte.. Genome Biol.

[41] Drews GN, Wang D, Steffen JG, Schumaker KS, Yadegari R (2011). Identification of genes expressed in the angiosperm female gametophyte.. J Exp Bot.

[42] Honys D, Twell D (2004). Transcriptome analysis of haploid male gametophyte development in *Arabidopsis*.. Genome Biol.

[43] Pina C, Pinto F, Feijó JA, Becker JD (2005). Gene family analysis of the *Arabidopsis* pollen transcriptome reveals biological implications for cell growth, division control, and gene expression regulation.. Plant Physiol.

[44] Wang Y, Zhang WZ, Song LF, Zou JJ, Su Z (2008). Transcriptome analyses show changes in gene expression to accompany pollen germination and tube growth in *Arabidopsis*.. Plant Physiol.

[45] Meinke D, Sweeney C, Muralla R (2009). Integrating the genetic and physical maps of *Arabidopsis thaliana*: Identification of mapped alleles of cloned essential (*EMB*) genes.. PLoS ONE.

[46] Müller AJ (1963). Embryonentest zum Nachweis rezessiver Letalfaktoren bei *Arabidopsis thaliana*.. Biol Zentralbl.

[47] Meinke DW (1982). Embryo-lethal mutants of *Arabidopsis thaliana*: Evidence for gametophytic expression of the mutant genes.. Theor Appl Genet.

[48] Berg M, Rogers R, Muralla R, Meinke D (2005). Requirement of aminoacyl-tRNA synthetases for gametogenesis and embryo development in *Arabidopsis*.. Plant J.

[49] Bryant N, Lloyd J, Sweeney C, Myouga F, Meinke D (2011). Identification of nuclear genes encoding chloroplast-localized proteins required for embryo development in *Arabidopsis*.. Plant Physiol.

[50] Muralla R, Sweeney C, Stepansky A, Leustek T, Meinke D (2007). Genetic dissection of histidine biosynthesis in *Arabidopsis*.. Plant Physiol.

[51] Onodera Y, Nakagawa K, Haag JR, Pikaard D, Mikami T (2008). Sex-biased lethality or transmission of defective transcription machinery in *Arabidopsis*.. Genetics.

[52] Teng C, Dong H, Shi L, Deng Y, Mu J (2008). Serine palmitoyltransferase, a key enzyme for de novo synthesis of sphingolipids, is essential for male gametophyte development in *Arabidopsis*.. Plant Physiol.

[53] Persson S, Paredez A, Carroll A, Palsdottir H, Doblin M (2007). Genetic evidence for three unique components in primary cell-wall cellulose synthase complexes in *Arabidopsis*.. Proc Natl Acad Sci USA.

[54] Ge X, Dietrich C, Matsuno M, Li G, Berg H (2005). An *Arabidopsis* aspartic protease functions as an anti-cell-death component in reproduction and embryogenesis.. EMBO Rep.

[55] Boavida LC, McCormick S (2007). Temperature as a determinant factor for increased and reproducible *in vitro* pollen germination in *Arabidopsis thaliana*.. Plant J.

[56] Mascarenhas JP (1989). The male gametophyte of flowering plants.. Plant Cell.

[57] Bayer M, Nawy T, Giglione C, Galli M, Meinnel T (2009). Paternal control of embryonic patterning in *Arabidopsis thaliana*.. Science.

[58] Holmes-Davis R, Tanaka CK, Vensel WH, Hurkman WJ, McCormick S (2005). Proteome mapping of mature pollen of *Arabidopsis thaliana*.. Proteomics.

[59] Noir S, Bräutigam A, Colby T, Schmidt J, Panstruga R (2005). A reference map of the *Arabidopsis thaliana* mature pollen proteome.. Biochem Biophys Res Commun.

[60] Sheoran IS, Sproule KA, Olson DJH, Ross ARS, Sawhney VK (2006). Proteome prolife and functional classification of proteins in *Arabidopsis thaliana* (Landsberg *erecta*) mature pollen.. Sex Plant Reprod.

[61] Zou J, Song L, Zhang W, Wang Y, Ruan S (2009). Comparative proteomic analysis of *Arabidopsis* mature pollen and germinated pollen.. J Integ Plant Biol.

[62] Grobei MA, Qeli E, Brunner E, Rehrauer H, Zhang R (2009). Deterministic protein inference for shotgun proteomics data provides new insights into *Arabidopsis* pollen development and function.. Genome Res.

[63] Chen C, Farmer AD, Langley RJ, Mudge J, Crow JA (2010). Meiosis-specific gene discovery in plants: RNA-Seq applied to isolated *Arabidopsis* male meiocytes.. BMC Plant Biol.

[64] Yang H, Lu P, Wang Y, Ma H (2011). The transcriptome landscape of *Arabidopsis* male meiocytes from high-throughput sequencing: The complexity and evolution of the meiotic process.. Plant J.

[65] Libeau P, Durandet M, Granier F, Marquis C, Berthomé R (2011). Gene expression profiling of *Arabidopsis* meiocytes.. Plant Biol.

[66] Schmidt A, Wuest SE, Vijverberg K, Baroux C, Kleen D (2011). Transcriptome analysis of the *Arabidopsis* megaspore mother cell uncovers the importance of RNA helicases for plant germline development.. PLoS Biol.

[67] Wuest SE, Vijverberg K, Schmidt A, Weiss M, Gheyselinck J (2010). *Arabidopsis* female gametophyte gene expression map reveals similarities between plant and animal gametes.. Curr Biol.

[68] Grimanelli D, Perotti E, Ramirez J, Leblanc O (2005). Timing of the maternal-to-zygotic transition during early seed development in maize.. Plant Cell.

[69] Meyer S, Scholten S (2007). Equivalent parental contribution to early plant zygotic development.. Curr Biol.

[70] Jahnke S, Scholten S (2009). Epigenetic resetting of a gene imprinted in plant embryos.. Curr Biol.

[71] Vielle-Calzada JP, Baskar R, Grossniklaus U (2000). Delayed activation of the paternal genome during seed development.. Nature.

[72] Weijers D, Geldner N, Offringa R, Jürgens G (2001). Early paternal gene activity in *Arabidopsis*.. Nature.

[73] Pillot M, Baroux C, Vazquez MA, Autran D, Leblanc O (2010). Embryo and endosperm inherit distinct chromatin and transcriptional states from the female gametes in *Arabidopsis*.. Plant Cell.

[74] Aw SJ, Hamamura Y, Chen Z, Schnittger A, Berger F (2010). Sperm entry is sufficient to trigger division of the central cell but the paternal genome is required for endosperm development in *Arabidopsis*.. Development.

[75] Raissig MT, Baroux C, Grossniklaus U (2011). Regulation and flexibility of genomic imprinting during seed development.. Plant Cell.

[76] Xiang D, Venglat P, Tibiche C, Yang H, Risseeuw E (2011). Genome-wide analysis reveals gene expression and metabolic network dynamics during embryo development in *Arabidopsis*.. Plant Physiol.

[77] Autran D, Baroux C, Raissig M, Lenormand T, Wittig M (2011). Maternal epigenetic pathways control parental contributions to *Arabidopsis* early embryogenesis.. Cell.

[78] Grossniklaus U, Vielle-Calzada JP, Hoeppner MA, Gagliano WB (1998). Maternal control of embryogenesis by *MEDEA*, a polycomb group gene in *Arabidopsis*.. Science.

[79] Kiyosue T, Ohad N, Yadegari R, Hannon M, Dinney J (1999). Control of fertilization-independent endosperm development by the *MEDEA* polycomb gene in *Arabidopsis*.. Proc Natl Acad Sci USA.

[80] Kinoshita T, Yadegari R, Harada JJ, Goldberg RB, Fischer RL (1999). Imprinting of the *MEDEA* polycomb gene in the *Arabidopsis* endosperm.. Plant Cell.

[81] Luo M, Bilodeau P, Koltunow A, Dennis ES, Peacock WJ (1999). Genes controlling fertilization-independent seed development in *Arabidopsis thaliana*.. Proc Natl Acad Sci USA.

[82] Choi Y, Gehring M, Johnson L, Hannon M, Harada JJ (2002). DEMETER, a DNA glycosylase domain protein, is required for endosperm gene imprinting and seed viability in *Arabidopsis*.. Cell.

[83] Muralla R, Chen E, Sweeney C, Gray JA, Dickerman A (2008). A bifunctional locus (*BIO1–BIO3*) required for biotin biosynthesis in *Arabidopsis*.. Plant Physiol.

[84] Meinke DW, Meinke LK, Showalter TC, Schissel AM, Mueller LA (2003). A sequence-based map of *Arabidopsis* genes with mutant phenotypes.. Plant Physiol.

